# Supplementary material for: CoproID predicts the source of coprolites and paleofeces using microbiome composition and host DNA content
Source: PeerJ. 2020 Apr 17;8:e9001. doi: 10.7717/peerj.9001 (PMC7169968; doi:10.7717/peerj.9001)
Supplement: Supplemental Information 1 [file peerj-08-9001-s001.pdf]

Supplementary material for the article "*CoproID* predicts the source of coprolites and paleofeces using microbiome composition and host DNA content"

1 Supplementary note

1.1 Preservation of coprolites

Taphonomic conditions affect the preservation potential of coprolites. These fall into two major categories: biological and sedimentological. To provide a taphonomic guide for molecular biologists working with coprolites, a reanalysis of coprolites from key sites in the desert west of North America was recently undertaken (Reinhard et al., 2019), and two common sedimentological factors affecting coprolites (even in desert environments) were identified: dry infiltration of sand and precipitation of calcium-bearing moisture. dry infiltration of sand into samples can occur in horizontally deep caves, and percolation of calcium-bearing moisture is more common in open sites and at the entrances to rockshelters. Both factors can result in the complete or partial mineralization of coprolites. In dry deposits, beetles are also known to infest coprolites, and sand from the surrounding sediment matrix can become compressed into the coprolites.

Flies, beetles, mites and fungi are visible biological taphonomic agents (Reinhard et al., 2019). These decomposers are especially abundant in dense deposits of paleofeces in ancient latrines. In such latrines, the feces remain moist during the full duration of use, perhaps for decades. The communities of decomposers that establish under such conditions are strongly detrimental to the preservation of the original intestinal material. In contrast, individual fecal specimens deposited in midden (trash) contexts of short-term use often exhibit far greater preservation, especially if the fecal material is dispersed among botanical waste such as leaves, fiber masses, or cordage.

A recent review of coprolite taphonomy provides some guidelines for identifying samples with enhanced preservation potential (Reinhard et al., 2019). Flies, nematodes and especially mites are associated with poor fecal preservation at the microscopic level (Reinhard et al., 2019). These decomposers are dependent on moisture availability and microbial processes of decomposition. Visual evidence of these types of decomposers signals poor preservation potential.

Of the paleofeces analyzed in this study, only the coprolites from La Cueva de los Chiquitos Muertos, Rio Zape, Durango Mexico (Zape) have undergone a formal taphonomic

assessment. From the preliminary excavation report (Brooks et al., 1962), field excavation notes were reviewed in 2019 to understand the distribution of coprolites within the cave. The Zape coprolites were dispersed in midden deposits up to 35 feet from the front of the cave. Among the midden contents, the coprolites were recovered together with the dessicated remains of maize, agave quids (expectorated agave fibers), beans, nuts, and a variety of other wild and domesticated plants (Brooks et al., 1962). The deposit covered an area 20 feet in breadth, ten feet in width and two feet in depth, and from this, it is inferred that the materials were deposited into a broad and open area within the cave. Such shallow deposits spread over a large area are known as "sheet trash." These conditions are optimal for long-term preservation, as the shallow, open-air exposure facilitates rapid drying in this arid region. The deposits, being spatially distant from the front of the cave, were protected from ambient moisture. However, they were infested with spider beetles. Such beetles prefer dry, organic substrates, and many coprolites displayed perforations left by spider beetles. It is noteworthy that these were the only decomposers identified at the site.

1.2 How do we tell human from animal?

Since the 1970s, determining the biological origin of coprolites has been a focused area of research (Reinhard, 2017). Previously, only coprolites associated with skeletons or mummies could be assured a human origin, although archaeological context can also provide strong support. For example, coprolites from latrines are very likely to be human, especially when the architectural structure prohibits animal entry. However, host species determination can be less clear when coprolites are associated with trash deposits. Chame (2003) published a guide to the morphology of mammal feces and argued that scat morphology is distinct enough between species to separate human and non-human coprolites. However, dogs are clearly an exception to this system. Because of canid scatophagy, dog coprolites can closely resemble those of humans both at a macroscopic and a microscopic level.

Since the first scientific assessment of coprolites (Fry, 1977), a variety of methods have been developed to help separate human from dog samples. These methods are applied during processing and have been recently reviewed and updated (Bryant and Dean, 2006; Bryant and Reinhard, 2012; Reinhard, 2017; Reinhard and Bryant, 2008; Taylor et al., 2019). The morphology of canid coprolites is often distinct with a desiccated mucous exterior and containing evidence of hair, intact small animal bones, large splinters of long bone, cordage, cobs, pebbles, and other extraneous cave material. The rehydration stage of analysis can be insightful as researchers since Fry (1977) repeated noted that human coprolites turned the rehydration solution dark brown or opaque black, while the rehydration solution of nonhuman coprolites typically remained clear or became yellow. Most recently, Chame and her colleagues (Ferreira et al., 1989) tested feces from twenty-two South American mammalian species and found that only four species produced a dark brown or black opaque reaction upon rehydration. During the rehydration process of dog feces, the mucous also rehydrates and becomes visible on the coprolite exterior.

Reinhard and Bryant (1992) have recommended that coprolites of human origin should be based on the analysis of coprolite components. Coprolites of canine origin typically contain masses of short, nibbled dog hairs and odd inclusions, such as fragments of clothing and rope. This approach was recently augmented with the application of scanning electron microscopy (SEM) (Reinhard et al., 2019). With the aid of SEM, the details of structures such as lignin, cellulose, starch, calcium oxalate, phytoliths, and plant sporopollenin were observed, which can help identify plant remains. Finally, certain parasites are host specific and can be used to infer host species. The delicate eggs of human pinworm (Enterobius vermicularis), for example, should only be present in human coprolites, as supported by the helminth Host-Parasite Catalogue produced by the Natural History Museum, London. Since 1922, the Host Parasite Catalogue records each host-parasite record from the scientific literature, including both true infections and false infections. A review of 70,000 references (Reinhard, 2017) showed that E. vermicularis eggs had never been found in dog excreta, nor that of any non-primates. Thus, its presence in coprolite material should be considered as an additional diagnostic for human feces.

References

Brooks, R. H., Kaplan, L., Cutler, H. C., and Whitaker, T. W. (1962). Plant material from a cave on the rio zape, durango, mexico. *American Antiquity*, 27(3):356–369.

Bryant, V. M. and Dean, G. W. (2006). Archaeological coprolite science: the legacy of eric o. callen (1912–1970). *Palaeogeography, Palaeoclimatology, Palaeoecology*, 237(1):51–66.

Bryant, V. M. and Reinhard, K. J. (2012). Coprolites and archaeology: the missing links in understanding human health. *Vertebrate coprolites: New Mexico museum of natural history and science, bulletin*, 57:379–387.

Chame, M. (2003). Terrestrial mammal feces: A morphometric summary and description. *Memórias do Instituto Oswaldo Cruz*, 98 Suppl 1:71–94.

Ferreira, L. F., Araújo, A., Confalonieri, U., and Chame, M. (1989). Acanthocefalan eggs in animal coprolites from archaeological sites from brazil. *Memórias do Instituto Oswaldo Cruz*, 84(2):201–203.

Fry, G. F. (1977). *Analysis of Prehistoric Coprolites from Utah (Anthropological Papers; No. 97)*. University of Utah.

Reinhard, K. (2017). Reestablishing rigor in archaeological parasitology. *International journal of paleopathology*, 19:124–134.

Reinhard, K. and Bryant, V. (2008). Pathoecology and the future of coprolite studies. stodder awm, editor. reanalysis and reinterpretation in southwestern bioarchaeology. tempe.

Reinhard, K., Camacho, M., Geyer, B., Hayek, S., Horn, C., Otterson, K., and Russ, J. (2019). Imaging coprolite taphonomy and preservation. *Archaeological and Anthropological Sciences*, pages 1–19.

Reinhard, K. J. and Bryant, V. M. (1992). Coprolite analysis: a biological perspective on archaeology. *Archaeological method and theory*, 4:245–288.

Taylor, A., Hutson, J. M., Bryant, V. M., and Jenkins, D. L. (2019). Dietary items in early to late holocene human coprolites from paisley caves, oregon, usa. *Palynology*, pages 1–12.

2 Supplementary tables

Table S1: Host and Total read count in reference modern gut shotgun metagenomics samples

sample	host_read_count	all_read_count	percentage_host_DNA	organism
E5	175600	11487470	1.53	WHU
F4	183259	9696234	1.89	WHU
E2	214203	8327324	2.57	WHU
E1	227278	10907632	2.08	WHU
B10	375059	11695702	3.21	WHU
C3	51792	10324844	0.5	WHU
B7	423399	11698532	3.62	WHU
A1	153528	10803284	1.42	WHU
E12	180817	12377546	1.46	WHU
D3	254238	11408122	2.23	WHU
C2	43231	9075014	0.48	WHU
D7	225207	11510432	1.96	WHU
D1	269617	12033292	2.24	WHU
B2	112611	13075492	0.86	WHU
A2	120257	10901438	1.1	WHU
A6	160317	12356964	1.3	WHU
A8	273006	10723706	2.55	WHU
C10	215235	13017016	1.65	WHU
C9	136464	10845174	1.26	WHU
B8	340796	10653408	3.2	WHU
B4	166426	10508250	1.58	WHU
A3	145562	12421768	1.17	WHU
D12	237623	9704844	2.45	WHU
C11	284235	11279754	2.52	WHU
E11	192428	11743308	1.64	WHU
A5	215066	11882440	1.81	WHU
B1	63371	15456544	0.41	WHU

Table S1 continued from previous page

sample	host_read_count	all_read_count	percentage_host_DNA	organism
A11	199370	9271072	2.15	WHU
C1	184712	11074722	1.67	WHU
C12	238833	10917346	2.19	WHU
D6	386640	11700012	3.3	WHU
F1	262879	10183936	2.58	WHU
B11	298888	10967064	2.73	WHU
B5	126996	12667796	1.0	WHU
D11	155294	8180156	1.9	WHU
B3	133929	11241482	1.19	WHU
D5	243972	11283954	2.16	WHU
A9	186274	8874688	2.1	WHU
C4	42364	9825904	0.43	WHU
D2	177084	11314076	1.57	WHU
D9	143234	10521106	1.36	WHU
E3	150478	10696592	1.41	WHU
E9	197977	9446418	2.1	WHU
C7	180169	15386204	1.17	WHU
F3	150425	11413220	1.32	WHU
A4	110957	10818038	1.03	WHU
E6	440959	11818510	3.73	WHU
D8	196516	10573060	1.86	WHU
C6	137728	14126344	0.97	WHU
bftm2301ext33	55095	18796990	0.29	NWHR
bftm070332	49029	26437410	0.19	NWHR
bftm030236	77452	27571762	0.28	NWHR
bftm2401ext31	7908	28078268	0.03	NWHR
bftm280135	4046109	31803446	12.72	NWHR
bftm130122	12870	41265242	0.03	NWHR
bftm0401ecol39	34853	42149188	0.08	NWHR
bftm230440	2028606	42879336	4.73	NWHR
bftm1502ecol15	39315	51388140	0.08	NWHR
bftm040212	11484	40581568	0.03	NWHR
bftm2103ext32	48689	46270246	0.11	NWHR
bftm140428	40617	55616436	0.07	NWHR
bftm180134	21821	25888008	0.08	NWHR
bftm110237	19889	43720724	0.05	NWHR
bftm1003ext22	12289	47524028	0.03	NWHR
bftm0803ext30	8469	18437208	0.05	NWHR
bftm100113	1309446	100223950	1.31	NWHR
bftm190220	31017	69358310	0.04	NWHR
bftm270223	40999	46300184	0.09	NWHR
bftm220210	26878	85778526	0.03	NWHR
bftm0202ext20	13721	49753554	0.03	NWHR

Table S1 continued from previous page

sample	host_read_count	all_read_count	percentage_host_DNA	organism
bftm0601ext18	7489	26092968	0.03	NWHR
bftm140127	293440	104080352	0.28	NWHR
bftm2801ext35	1708342	16746372	10.2	NWHR
bftm1002ecol4	30725	52397704	0.06	NWHR
bftm1303ext17	6928	28080332	0.02	NWHR
bftm140341	157727	83257562	0.19	NWHR
bftm2204ext27	367707	56935542	0.65	NWHR
bftm30016	106606	74694944	0.14	NWHR
bftm050238	114780	32861610	0.35	NWHR
bftm1104ecol11	7688	52221098	0.01	NWHR
bftm060125	20622	54179132	0.04	NWHR
bftm0701ecol12	17077	39086588	0.04	NWHR
bftm2101ext26	2557108	54682456	4.68	NWHR
bftm2601ext24	143719	58945896	0.24	NWHR
bftm3003ext25	228585	28016386	0.82	NWHR
bftm09037	15466	43494168	0.04	NWHR
bftm0604ecol14	26157	43001534	0.06	NWHR
bftm110126	16884	37752822	0.04	NWHR
bftm030111	72776	58457354	0.12	NWHR
bftm290124	12148	67245490	0.02	NWHR
bftm1704ext28	19128	48362604	0.04	NWHR
bftm150339	52807	56123024	0.09	NWHR
bftm2704ext23	194657	43787378	0.44	NWHR
bftm1601ecol9	106670	73952044	0.14	NWHR
bftm1104ext21	14441	46757422	0.03	NWHR
bftm3004ext36	83706	30880108	0.27	NWHR
bftm26043	241501	40648682	0.59	NWHR
bftm2304ext34	409429	10953162	3.74	NWHR
bftm2301ecol5	260661	74217736	0.35	NWHR
bftm1603ecol2	80129	54867598	0.15	NWHR
bftm120433	62164	19740812	0.31	NWHR
bftm080321	19267	48315608	0.04	NWHR
bftm2504ecol13	1344123	52388322	2.57	NWHR
bftm0101ecol	11690	62461836	0.02	NWHR
bftm220116	160165	70274880	0.23	NWHR
bftm1402ext19	27382	41748458	0.07	NWHR
bftm17021	149345	30179114	0.49	NWHR
bftm1701ecol6	11063	86439714	0.01	NWHR
bftm150117	35070	77016038	0.05	NWHR
bftm22034	165046	60784258	0.27	NWHR
bftm0902ecol40	155633	30088172	0.52	NWHR
bftm01028	10943	44433538	0.02	NWHR
bftm2501ecol7	37260	53232538	0.07	NWHR

Table S1 continued from previous page

sample	host_read_count	all_read_count	percentage_host_DNA	organism
bftm200318	88945	56072148	0.16	NWHR
bftm080215	16237	76984636	0.02	NWHR
bftm200129	212498	73008450	0.29	NWHR
bftm010319	28985	63373402	0.05	NWHR
bftm2002ext29	4930107	53649054	9.19	NWHR
ERR1914445	608	5149226	0.01	Dog
ERR1915141	199	3533432	0.01	Dog
ERR1916240	2398	2959452	0.08	Dog
ERR1915117	1224	4438944	0.03	Dog
ERR1913401	2085	3861284	0.05	Dog
ERR1915610	3612	3616066	0.1	Dog
ERR1915410	2647	3802428	0.07	Dog
ERR1916166	893	4657978	0.02	Dog
ERR1914224	3162	7968330	0.04	Dog
ERR1913830	4042	4956394	0.08	Dog
ERR1915971	3066	4209920	0.07	Dog
ERR1916218	865	4191090	0.02	Dog
ERR1916299	1363	3491562	0.04	Dog
ERR1915335	1355	4116570	0.03	Dog
ERR2402762	18498	4063404	0.46	Dog
ERR1914696	15239	4051532	0.38	Dog
ERR1914207	7208	4447510	0.16	Dog
ERR1914085	3503	4028044	0.09	Dog
ERR1913614	799	4337814	0.02	Dog
ERR1913472	37622	4346212	0.87	Dog
ERR1915751	454	3857096	0.01	Dog
ERR1916229	2312	2731206	0.08	Dog
ERR1915582	253	4691122	0.01	Dog
ERR1914944	16628	3581252	0.46	Dog
ERR2402765	18942	4198878	0.45	Dog
ERR1916302	1495	3500200	0.04	Dog
ERR1915103	1230	4197006	0.03	Dog
ERR1913556	1161	4701010	0.02	Dog
ERR1914307	1630	4460470	0.04	Dog
ERR1915539	3850	5618600	0.07	Dog
ERR1915309	1978	4852570	0.04	Dog
ERR1915204	479	3744596	0.01	Dog
ERR1914185	6159	3697256	0.17	Dog
ERR1915816	5834	3822118	0.15	Dog
ERR1913391	10689	4830492	0.22	Dog
ERR1914332	652	3360198	0.02	Dog
ERR1915022	263	3385498	0.01	Dog
ERR1914439	540	4704484	0.01	Dog

Table S1 continued from previous page

sample	host_read_count	all_read_count	percentage_host_DNA	organism
ERR1914302	1499	3674014	0.04	Dog
ERR1914320	1873	4628300	0.04	Dog
ERR1913524	1089	2602736	0.04	Dog
ERR1915317	1060	3177654	0.03	Dog
ERR1913948	34470	5574974	0.62	Dog
ERR1914316	1907	4707448	0.04	Dog
ERR2402795	11041	7701058	0.14	Dog
ERR1916189	403	4469802	0.01	Dog
ERR1913963	37461	6034196	0.62	Dog
ERR1915826	5715	3708276	0.15	Dog
ERR1914111	2646	5868374	0.05	Dog
ERR1916042	1166	4125478	0.03	Dog
ERR1915011	165	2450928	0.01	Dog
ERR1915380	1104	5994652	0.02	Dog
ERR1913847	4226	5259340	0.08	Dog
ERR1915423	2539	3681956	0.07	Dog
ERR1913542	1089	4522812	0.02	Dog
ERR1913392	2317	3933230	0.06	Dog
ERR1915782	716	3017150	0.02	Dog
ERR1915499	1215	3880756	0.03	Dog
ERR1915611	3642	3714362	0.1	Dog
ERR1915376	1025	5993272	0.02	Dog
ERR1914041	3372	3524712	0.1	Dog
ERR1914553	357	3517282	0.01	Dog
ERR1914438	453	3627364	0.01	Dog
ERR1916315	1563	3665318	0.04	Dog
ERR1913675	1456	4214100	0.03	Dog
ERR1915420	2338	3481940	0.07	Dog
ERR1915225	3930	3020966	0.13	Dog
ERR1914208	3053	7614094	0.04	Dog
ERR1914674	1710	3840494	0.04	Dog
ERR1913829	3673	4821074	0.08	Dog
ERR1915012	153	2369958	0.01	Dog
ERR1913953	35031	5498102	0.64	Dog
ERR2402780	10542	7265952	0.15	Dog
ERR1914424	632	4867382	0.01	Dog
ERR1915578	259	4529930	0.01	Dog
ERR1916319	1637	3652672	0.04	Dog
ERR1915100	1240	4141642	0.03	Dog
ERR2402772	18280	4026780	0.45	Dog
ERR2402760	18150	4039828	0.45	Dog
ERR1914932	15164	3287538	0.46	Dog
ERR1913663	1303	4040726	0.03	Dog

Table S1 continued from previous page

sample	host_read_count	all_read_count	percentage_host_DNA	organism
ERR1914441	668	4917562	0.01	Dog
ERR1914215	2848	7596536	0.04	Dog
ERR1915229	3875	3003368	0.13	Dog
ERR1915197	445	3757648	0.01	Dog
ERR1915363	1068	5700780	0.02	Dog
ERR1916170	930	4896070	0.02	Dog
ERR1916139	1827	4661524	0.04	Dog
ERR1913608	873	4331384	0.02	Dog
ERR1915231	4195	3172694	0.13	Dog
ERR1913387	10563	4771292	0.22	Dog
ERR1913805	4669	5005990	0.09	Dog
ERR1916312	1574	3666764	0.04	Dog
ERR1915253	888	2681768	0.03	Dog
ERR1914107	2628	5828016	0.05	Dog
ERR1916180	374	4176872	0.01	Dog
ERR1914213	2976	7529110	0.04	Dog
ERR1913944	35628	5713266	0.62	Dog
ERR1914430	561	4651614	0.01	Dog
ERR1913400	2197	3848794	0.06	Dog
ERR1914987	17358	6702566	0.26	Dog
ERR1914088	2503	5480602	0.05	Dog
ERR1915765	577	3999508	0.01	Dog
ERR1914605	3411	5270774	0.06	Dog
ERR1913527	1392	3144084	0.04	Dog
ERR1915320	1193	3635468	0.03	Dog
ERR1915102	1360	4220982	0.03	Dog
ERR1915186	325	5789872	0.01	Dog
ERR1913947	34769	5680650	0.61	Dog
ERR1915745	528	3839130	0.01	Dog
ERR1915563	3920	4000478	0.1	Dog
ERR1914040	3115	3600712	0.09	Dog
ERR1913950	35171	5718084	0.62	Dog
ERR1916193	383	4427438	0.01	Dog
ERR1915453	342	3901528	0.01	Dog
ERR1914997	17044	6507116	0.26	Dog
ERR1914292	1345	3294266	0.04	Dog
ERR1915122	216	3518714	0.01	Dog
ERR1916182	435	4189138	0.01	Dog
ERR1915558	3896	3891880	0.1	Dog
ERR1914985	17576	6617046	0.27	Dog
ERR1914349	702	3639940	0.02	Dog
ERR1916187	471	4527058	0.01	Dog
ERR1914611	3202	5193784	0.06	Dog

Table S1 continued from previous page

sample	host_read_count	all_read_count	percentage_host_DNA	organism
ERR1915438	372	3720340	0.01	Dog
ERR1913555	1133	4746178	0.02	Dog
ERR1913372	10214	4489848	0.23	Dog
ERR1915094	851	5057952	0.02	Dog
ERR1915048	7115	4014060	0.18	Dog
ERR1915455	402	3885130	0.01	Dog
ERR1915529	3721	5474540	0.07	Dog
ERR1913389	10596	4813572	0.22	Dog
ERR1915571	4138	4134088	0.1	Dog
ERR1914196	4277	2525784	0.17	Dog
ERR1915757	513	3786956	0.01	Dog
ERR1914667	1541	3685008	0.04	Dog
ERR1914082	3314	3859180	0.09	Dog
ERR1915293	1756	4254094	0.04	Dog
ERR1915064	7349	4220568	0.17	Dog
ERR1916078	1252	5512918	0.02	Dog
ERR1914750	1693	4200270	0.04	Dog
ERR1913815	5349	5771454	0.09	Dog
ERR1913971	10729	5552042	0.19	Dog
ERR1915838	5895	4016142	0.15	Dog
ERR1915382	988	5989274	0.02	Dog
ERR1913780	4205	6474574	0.06	Dog
ERR1915590	261	4385590	0.01	Dog
ERR1915612	3884	3611340	0.11	Dog
ERR1915140	218	3329108	0.01	Dog
ERR1915367	1013	5707360	0.02	Dog

Table S2: Sourcepredict source estimation of the Archaelogical samples

	**Canis_familiaris**	**Homo_sapiens**	**Soil**	**unknown**
AHP003	0.036	0.017	0.521	0.426
ZSM005	0.05	0.922	0.014	0.014
ZSM002	0.05	0.922	0.014	0.014
ZSM029	0.05	0.922	0.014	0.014
BRF001	0.036	0.017	0.529	0.418
ZSM025	0.05	0.922	0.014	0.014
AHP002	0.041	0.019	0.595	0.345
DRL001	0.034	0.016	0.492	0.458
ZSM028	0.05	0.922	0.014	0.014
ZSM023	0.05	0.922	0.014	0.014
LEI010	0.036	0.017	0.524	0.423
ECO004	0.034	0.016	0.491	0.459
AHP001	0.032	0.015	0.469	0.484
AHP004	0.527	0.106	0.009	0.358
MLP001	0.031	0.015	0.455	0.499
YRK001	0.949	0.023	0.014	0.014
CMN001	0.033	0.016	0.486	0.465
ZSM031	0.05	0.922	0.014	0.014
ZSM027	0.05	0.922	0.014	0.014
CBA001	0.036	0.017	0.526	0.421

Table S3: Host (nbp) and Metagenomic (Sourcepredict) compontent of the coproID prediction

sample	nbp-human	nbp-dog	metagenomic-human	metagenomic-dog	coproID-dog	coproID_human
AHP001	0.002	0.998	0.015	0.032	0.032	0.0
AHP002	0.001	0.999	0.019	0.041	0.041	0.0
AHP003	0.001	0.999	0.017	0.036	0.036	0.0
AHP004	0.009	0.991	0.106	0.527	0.522	0.001
BRF001	0.024	0.976	0.017	0.036	0.035	0.0
CBA001	0.125	0.875	0.017	0.036	0.032	0.002
CMN001	0.286	0.714	0.016	0.033	0.024	0.004
DRL001	0.16	0.84	0.016	0.034	0.028	0.003
ECO004	0.539	0.461	0.016	0.034	0.016	0.008
LEI010	0.365	0.635	0.017	0.036	0.023	0.006
MLP001	0.234	0.766	0.015	0.031	0.024	0.003
YRK001	0.003	0.997	0.023	0.949	0.946	0.0
ZSM002	0.002	0.998	0.922	0.05	0.05	0.002
ZSM005	0.87	0.13	0.922	0.05	0.007	0.802
ZSM023	0.002	0.998	0.922	0.05	0.05	0.002
ZSM025	0.639	0.361	0.922	0.05	0.018	0.589
ZSM027	0.54	0.46	0.922	0.05	0.023	0.497
ZSM028	0.544	0.456	0.922	0.05	0.023	0.501
ZSM029	0.004	0.996	0.922	0.05	0.05	0.003
ZSM031	0.586	0.414	0.922	0.05	0.021	0.54

Table S4: Raw sequencing read counts for Archaelogical samples

Sample	Unique Reads	Duplicate Reads
ZSM002_R1	37111676	7858482
ZSM002_R2	37248619	7721539
ZSM005_R1	49004175	1206423
ZSM005_R2	49069836	1140762
ZSM023_R1	9823755	227346
ZSM023_R2	9829938	221163
ZSM025_R1	23410382	1481795
ZSM025_R2	23453167	1439010
ZSM027_R1	19924780	465551
ZSM027_R2	19945920	444411
ZSM028_R1	65372773	1316675
ZSM028_R2	65465962	1223486
ZSM029_R1	22163035	1197136
ZSM029_R2	22183570	1176601
ZSM031_R1	26261987	1871175
ZSM031_R2	26300043	1833119
AHP001_R1	19140976	365830
AHP001_R2	19143473	363333
AHP002_R1	27574180	569839
AHP002_R2	27594652	549367
AHP003_R1	18974980	360583
AHP003_R2	18977042	358521
AHP004_R1	19634617	361873
AHP004_R2	19638201	358289
BRF001S0_R1	9442462	185962
BRF001S0_R2	9444872	183552
CBA001S0_R1	8826456	153236
CBA001S0_R2	8828636	151056
CMN001_R1	8015084	127151
CMN001_R2	8013821	128414
DRL001S0_R1	7609890	138037
DRL001S0_R2	7618507	129420
ECO004_R1	9565407	158405
ECO004_R2	9565984	157828
LEI010S0_R1	821253	6683465
LEI010S0_R2	1063210	6441508
MLP001S0_R1	1115199	6208693
MLP001S0_R2	1295090	6028802
YRK001S0_R1	10830480	534437
YRK001S0_R2	10845506	519411

Metagenome source	Food production	Other	N	Source
Homo sapiens - USA (Boston)	WHU	long term (>50 years) type 1 diabetes	49	This study
Homo sapiens - Burkina Faso	NWHR	-	69	This study

Table S5: Modern human reference microbiome datasets for gut host DNA estimation

3 Supplementary figures

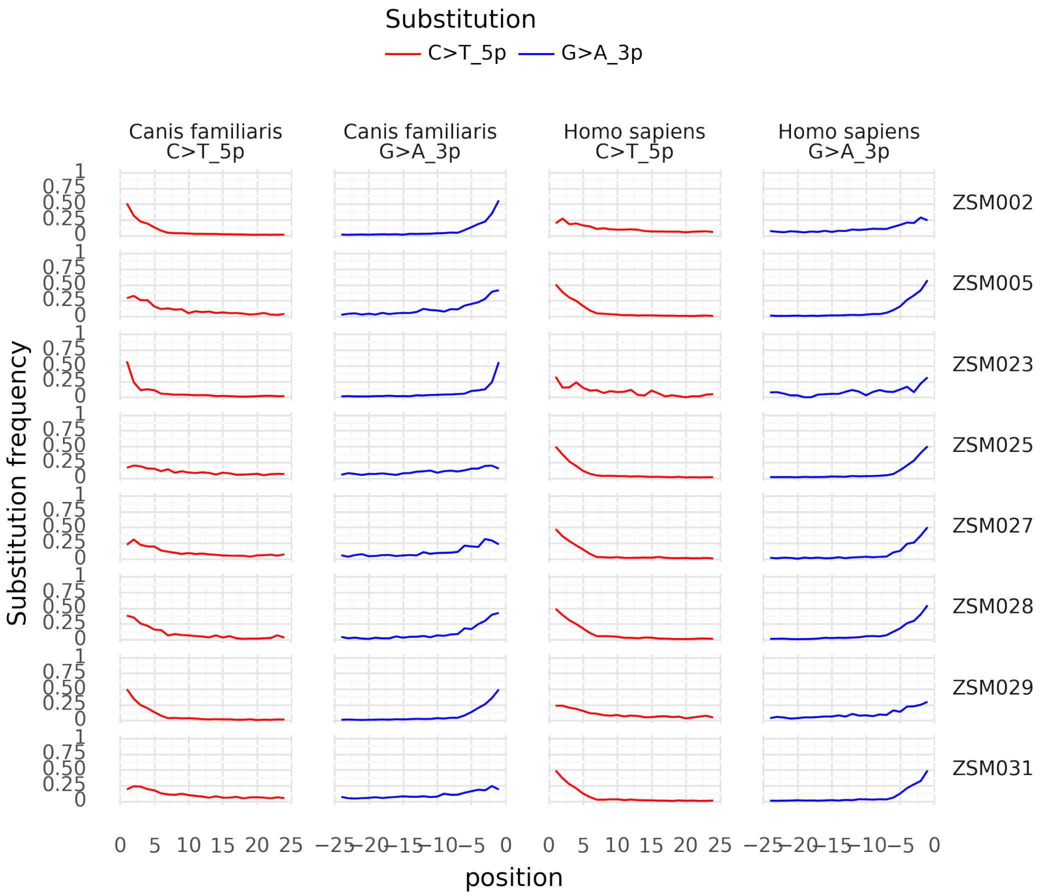

Figure S1: **Damage plot for non-UDG treated samples**

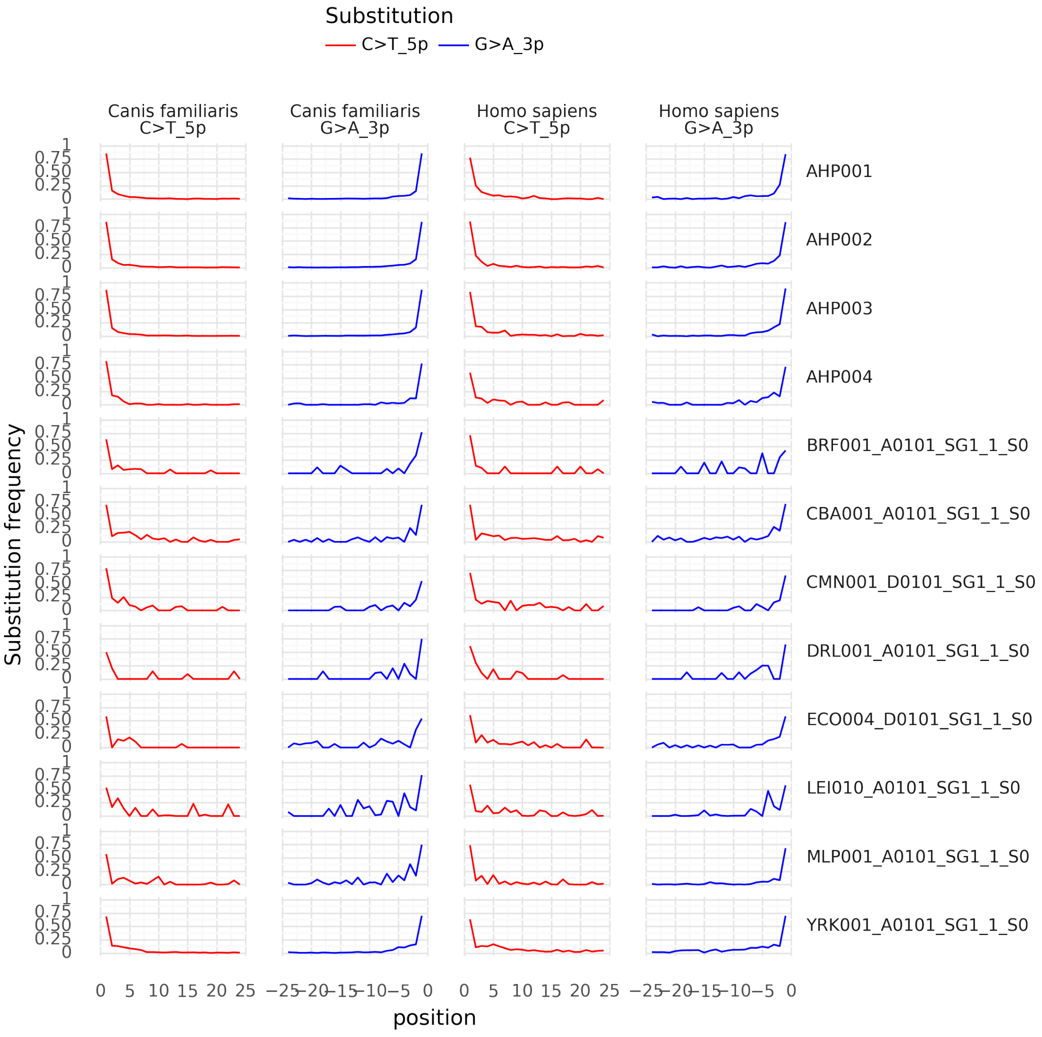

Figure S2: **Damage plot for UDG-half treated samples**

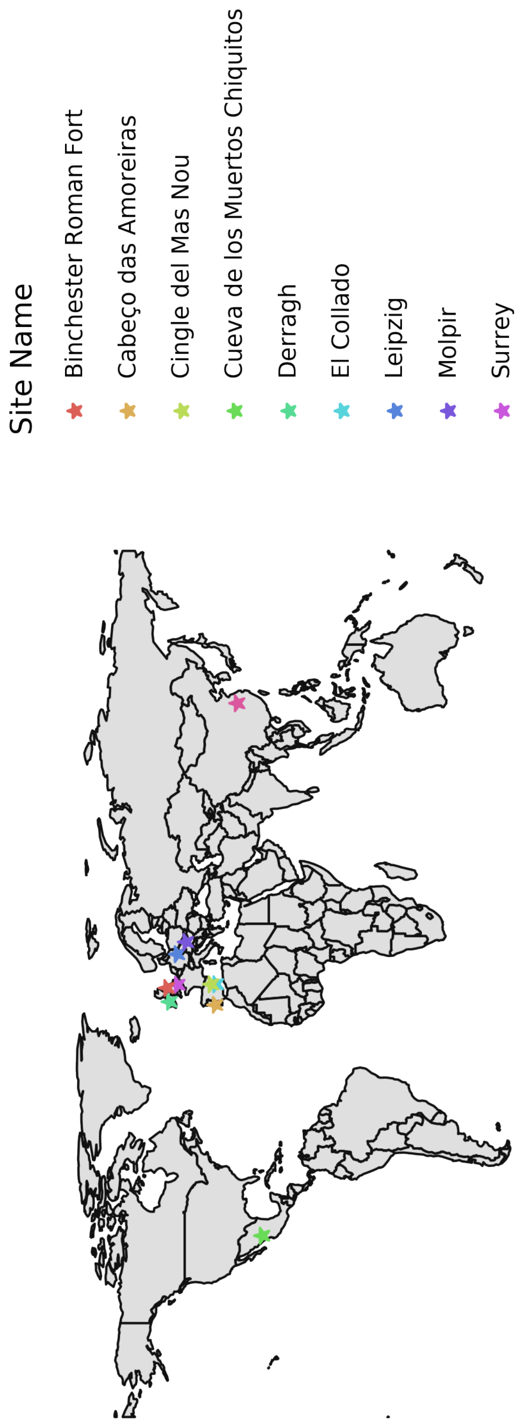

Figure S3: **Geographical distribution of Archaelogical sites**
